# Supplementary material for: Reward regulation in plant–frugivore networks requires only weak cues
Source: Nat Commun. 2018 Nov 16;9:4838. doi: 10.1038/s41467-018-07362-z (PMC6240120; doi:10.1038/s41467-018-07362-z)
Supplement: Supplementary file 1 — Supplementary Information [file 41467_2018_7362_MOESM1_ESM.pdf]

Supplementary Information

Reward regulation in plant–frugivore networks requires only weak cues

Albrecht et al.

Supplementary Table 1. Summary of sampling effort across the ten networks in each site and season.

Site	Season	Observation hours [h]	Plant species richness	Animal species richness	Visitation rate per hour	Fruit consumption rate per hour	Total number of recorded visits	Total number of estimated fruit consumption events
DE	DJF	294.0	10	7	34.53	117.67	430	1165
DE	JJA	444.0	19	22	117.64	426.56	2100	8293
DE	MAM	75.0	3	6	10.51	45.58	272	1127
DE	SON	627.0	21	25	103.12	402.75	3550	21204
GB	DJF	469.3	18	15	231.95	1157.72	6273	30498
GB	JJA	439.9	22	18	93.43	363.62	2391	9225
GB	MAM	241.1	7	10	21.59	122.69	1840	12841
GB	SON	915.6	23	23	149.50	773.96	10036	58870
PL	JJA	2166.0	13	26	29.53	95.17	4852	13654
PL	SON	768.0	8	13	10.93	37.06	1222	2712

Given are for each network the study site on which observations were conducted (PL, Poland, Albrecht et al.[1]; DE, Germany, Stiebel & Bairlein[2]; GB, Great Britain, Snow & Snow[3]), the season during which observations were conducted (MAM, March to May; JJA, June to August; SON, September to November; DJF, December to February), the number of observation hours (h), the number of plant and animal species in the network, the total visitation and fruit consumption rate per hour in the network, as well as the total number of recorded visits and the estimated total number of fruits consumed in the network.

Supplementary Table 2. Variance explained by fixed and random effects in the Bayesian hierarchical structural equation model testing whether the fruit choice of frugivorous birds is mediated by fruit colour and whether the birds' mean intake of particular nutrients is related to their partner diversity, interaction strength and migratory behaviour (see Table 2 in main text).

	Source of variance					
Response variable	$r^2_{marginal}$	r^2_{phylo}	$r^2_{species}$	r^2_{site}	r^2_{time}	$r^2_{conditional}$
x	0.0025	0.014	0.017	0.38	0.34	0.75
y	0.015	0.043	0.023	0.26	0.44	0.78
z	0.022	0.031	0.018	0.29	0.24	0.6
a	0.17	0.037	0.088	0.1	0.25	0.65
Lipid	0.14	0.039	0.044	0.091	0.47	0.78
Sugar	0.091	0.018	0.053	0.31	0.16	0.63
Protein	0.041	0.021	0.0068	0.4	0.32	0.79
Anthocyanin	0.48	0.0042	0.0065	0.18	0.2	0.86

The structural equation model tested for direct and indirect effects of the partner diversity and interaction strength of frugivores in the networks and their migratory distance on the colour profile of consumed fruits (i.e., chromatic colour components (x, y, z) and the brightness (a) in avian colour space; see Methods) and on the mean intake of particular nutrients (i.e., lipid, sugar, protein, anthocyanin). The sample size was $n_{obs} = 165$ observations across $n_{species} = 43$ bird species, $n_{site} = 3$ study sites and $n_{time} = 4$ seasons. Animal phylogeny, species, site and season were included as random factors. Given are r^2-values for the marginal variance ($r^2_{marginal}$) explained by the fixed factors only, as well as the variance that is explained by each of the random factors (r^2_{phylo}, $r^2_{species}$, r^2_{site}, r^2_{time}), and the variance explained by the fixed and random factors combined ($r^2_{conditional}$).

Supplementary Table 3. Summary of Bayesian hierarchical structural equation model based on visitation rate per hour instead of fruit consumption rate per hour as 'interaction currency'.

Response ~ predictor	Effect (95% CI)	P	BF	Response ~ predictor	Effect (95% CI)	P	BF
x ~				Lipid intake ~			
(r_m^2 = 0.0029, r_c^2 = 0.74)				(r_m^2 = 0.16, r_c^2 = 0.78)			
Partner diversity	-0.02 (-0.2, 0.04)	0.25	-2.2	Partner diversity	0.01 (-0.07, 0.2)	0.25	-2.2
Interaction strength	-0.01 (-0.2, 0.06)	0.22	-2.6	Interaction strength	0.1 (0, 0.3)	0.69	1.6
Migratory distance	0.01 (-0.05, 0.1)	0.23	-2.5	Migratory distance	-0.002 (-0.1, 0.08)	0.19	-2.9
				x	0.003 (-0.09, 0.1)	0.19	-2.9
				y	0.3 (0.1, 0.4)	0.99	9.5*
				z	-0.01 (-0.2, 0.04)	0.22	-2.6
				a	0.5 (0.3, 0.6)	1.0	>15*
y ~				Sugar intake ~			
(r_m^2 = 0.013, r_c^2 = 0.78)				(r_m^2 = 0.089, r_c^2 = 0.66)			
Partner diversity	-0.09 (-0.3, 0)	0.54	0.30	Partner diversity	0.08 (-0.01, 0.4)	0.45	-0.41
Interaction strength	-0.03 (-0.2, 0.07)	0.34	-1.4	Interaction strength	-0.04 (-0.3, 0.04)	0.34	-1.3
Migratory distance	0.06 (-0.04, 0.3)	0.41	-0.74	Migratory distance	0.003 (-0.08, 0.1)	0.19	-2.9
				x	-0.1 (-0.6, 4e-04)	0.51	0.064
				y	-0.07 (-0.4, 0.01)	0.42	-0.60
				z	-0.05 (-0.5, 0.1)	0.36	-1.2
				a	-0.4 (-0.6, -0.2)	1.0	15*
z ~				Protein intake ~			
(r_m^2 = 0.0088, r_c^2 = 0.58)				(r_m^2 = 0.043, r_c^2 = 0.80)			
Partner diversity	0.005 (-0.08, 0.1)	0.22	-2.6	Partner diversity	-0.05 (-0.3, 0.02)	0.38	-0.97
Interaction strength	0.006 (-0.09, 0.1)	0.22	-2.6	Interaction strength	-0.01 (-0.2, 0.07)	0.24	-2.4
Migratory distance	-0.06 (-0.3, 0.002)	0.41	-0.70	Migratory distance	-0.004 (-0.1, 0.07)	0.18	-3.0
				x	0.1 (-0.005, 0.5)	0.48	-0.14
				y	0.3 (0.1, 0.5)	0.99	10*
				z	0.06 (-0.09, 0.4)	0.37	-1.1
				a	0.02 (-0.05, 0.2)	0.27	-2.0
a ~				Anthocyanin intake ~			
(r_m^2 = 0.14, r_c^2 = 0.64)				(r_m^2 = 0.43, r_c^2 = 0.88)			
Partner diversity	0.3 (0.02, 0.5)	0.98	7.5*	Partner diversity	0.08 (0, 0.2)	0.69	1.6
Interaction strength	0.03 (-0.05, 0.3)	0.27	-2.0	Interaction strength	0.01 (-0.05, 0.1)	0.24	-2.3
Migratory distance	-0.3 (-0.5, 0)	0.94	5.4*	Migratory distance	0.007 (-0.005, 0.09)	0.17	-3.2
				x	0.3 (0, 0.5)	0.94	5.5*
				y	0.4 (0.2, 0.5)	1.0	>15*
				z	0.5 (0.3, 0.7)	1.0	>15*
				a	-0.7 (-0.8, -0.6)	1.0	>15*

The structural equation model tested for direct and indirect effects of the partner diversity and interaction strength of frugivores in the networks and their migratory distance on the colour profile of consumed fruits (i.e., chromatic colour components (*x*, *y*, *z*) and the brightness (*a*) in avian colour space; see Methods) and on the mean intake of particular nutrients (i.e., lipid, sugar, protein, anthocyanin). The sample size was $n_{\text{obs}} = 165$ observations across $n_{\text{species}} = 43$ bird species, $n_{\text{site}} = 3$ study sites and $n_{\text{time}} = 4$ seasons. Animal phylogeny, species, site and season were included as random factors. Given are posterior means (with shrinkage), 95% credible intervals (CI), selection probabilities (P) and 2log$_e$(Bayes factor) (BF) as a measure of support for a given effect. BF-values < 2 indicate no support; values between 2 and 6 indicate positive support; values between 6 and 10 indicate strong support; and values > 10 indicate decisive support. Effects that were supported by the variable selection with BF > 2 are highlighted with an asterisk. The r^2 values depict the marginal (r_m^2) variance explained by fixed factors only as well as the conditional (r_c^2) variance explained by fixed and random factors combined[4]. Note that the results are virtually identical to the results based on fruit consumption rate per hour (see Table 2 in main text).

Supplementary Table 4. Summary of Bayesian hierarchical structural equation model in which the migratory distance outside the migration and pre-migration period was not set to zero (alternative model 1).

Response ~ predictor	Effect (95% CI)	P	BF	Response ~ predictor	Effect (95% CI)	P	BF
x ~				Lipid intake ~			
(r_m^2 = 0.0018, r_c^2 = 0.75)				(r_m^2 = 0.14, r_c^2 = 0.78)			
Partner diversity	-0.01 (-0.2, 0.04)	0.21	-2.7	Partner diversity	0.04 (-0.02, 0.2)	0.37	-1.1
Interaction strength	0.008 (-0.05, 0.1)	0.18	-3.0	Interaction strength	0.09 (0, 0.3)	0.59	0.69
Migratory distance	0.009 (-0.06, 0.1)	0.22	-2.6	Migratory distance	-0.002 (-0.1, 0.09)	0.18	-3.1
				x	7e-04 (-0.1, 0.1)	0.19	-2.9
				y	0.3 (0.1, 0.4)	0.99	8.6*
				z	-0.02 (-0.2, 0.03)	0.21	-2.6
				a	0.4 (0.3, 0.6)	1.0	15*
y ~				Sugar intake			
(r_m^2 = 0.02, r_c^2 = 0.78)				(r_m^2 = 0.094, r_c^2 = 0.62)			
Partner diversity	-0.1 (-0.3, 0)	0.63	1.1	Partner diversity	0.06 (-0.02, 0.3)	0.39	-0.92
Interaction strength	0.009 (-0.1, 0.2)	0.24	-2.3	Interaction strength	-0.03 (-0.3, 0.05)	0.28	-1.8
Migratory distance	0.1 (0, 0.4)	0.63	1.1	Migratory distance	0.007 (-0.1, 0.2)	0.21	-2.6
				x	-0.1 (-0.6, 0.01)	0.49	-0.08
				y	-0.08 (-0.4, 0.02)	0.46	-0.29
				z	-0.07 (-0.5, 0.1)	0.37	-1.0
				a	-0.4 (-0.6, -0.2)	1.0	>15*
z ~				Protein intake ~			
(r_m^2 = 0.012, r_c^2 = 0.6)				(r_m^2 = 0.041, r_c^2 = 0.79)			
Partner diversity	0.001 (-0.1, 0.1)	0.19	-2.9	Partner diversity	-0.04 (-0.3, 0.03)	0.33	-1.4
Interaction strength	-0.01 (-0.2, 0.06)	0.21	-2.6	Interaction strength	8e-04 (-0.1, 0.1)	0.18	-3.0
Migratory distance	-0.09 (-0.3, 0.005)	0.52	0.18	Migratory distance	-0.004 (-0.1, 0.07)	0.18	-3.0
				x	0.1 (0, 0.5)	0.53	0.22
				y	0.3 (0.1, 0.5)	0.99	8.4*
				z	0.05 (-0.1, 0.4)	0.37	-1.1
				a	0.02 (-0.04, 0.2)	0.27	-2.0
a ~				Anthocyanin intake ~			
(r_m^2 = 0.12, r_c^2 = 0.69)				(r_m^2 = 0.46, r_c^2 = 0.86)			
Partner diversity	0.4 (0.2, 0.5)	1.0	>15*	Partner diversity	0.08 (0, 0.2)	0.70	1.7
Interaction strength	-0.01 (-0.2, 0.06)	0.21	-2.7	Interaction strength	0.01 (-0.01, 0.1)	0.24	-2.3
Migratory distance	-0.3 (-0.5, 0)	0.93	5.3*	Migratory distance	0.003 (-0.02, 0.06)	0.13	-3.8
				x	0.4 (0.1, 0.6)	0.98	8.3*
				y	0.3 (0.2, 0.5)	1.0	>15*
				z	0.6 (0.3, 0.8)	1.0	>15*
				a	-0.7 (-0.9, -0.6)	1.0	>15*

The structural equation model tested for direct and indirect effects of the partner diversity and interaction strength of frugivores in the networks and their migratory distance on the colour profile of consumed fruits (i.e., chromatic colour components (*x*, *y*, *z*) and the brightness (*a*) in avian colour space; see Methods) and on the mean intake of particular nutrients (i.e., lipid, sugar, protein, anthocyanin). The sample size was $n_{\text{obs}} = 165$ observations across $n_{\text{species}} = 43$ bird species, $n_{\text{site}} = 3$ study sites and $n_{\text{time}} = 4$ seasons. Animal phylogeny, species, site and season were included as random factors. Given are posterior means (with shrinkage), 95% credible intervals (CI), selection probabilities (P) and $2\log_e$(Bayes factor) (BF) as a measure of support for a given effect. Effects that were supported by the variable selection with BF > 2 are highlighted with an asterisk. The r^2 values depict the marginal (r_m^2) variance explained by fixed factors only as well as the conditional (r_c^2) variance explained by fixed and random factors combined[4]. Note that the results are consistent with the results based on the model in which the migratory distance outside the migration and pre-migration period was set to zero (see Table 2 in main text).

Supplementary Table 5. Summary of Bayesian hierarchical structural equation model including period (migration versus non-migration) and its interaction with migratory distance (alternative model 2).

Response ~ predictor	Effect (95% CI)	P	BF	Response ~ predictor	Effect (95% CI)	P	BF
x ~				Lipid intake ~			
(r_m^2 = 0.0089, r_c^2 = 0.75)				(r_m^2 = 0.19, r_c^2 = 0.76)			
Partner diversity	-0.01 (-0.2, 0.05)	0.24	-2.3	Partner diversity	0.04 (-0.005, 0.2)	0.38	-1.0
Interaction strength	0.009 (-0.07, 0.2)	0.21	-2.7	Interaction strength	0.08 (0, 0.3)	0.55	0.41
Migratory distance	0.007 (-0.05, 0.1)	0.18	-3.0	Migratory distance	-0.003 (-0.1, 0.08)	0.19	-2.9
Period	-0.08 (-0.8, 0.5)	0.51	0.11	Period	0.2 (-0.4, 0.9)	0.61	0.86
Migratory dist. × period	-0.007 (-0.1, 0)	0.042	-2.3	Migratory dist. × period	-0.04 (-0.4, 0.2)	0.38	3.0*
				x	-0.01 (-0.2, 0.1)	0.26	-2.1
				y	0.3 (0.09, 0.4)	1.0	>15*
				z	-0.06 (-0.3, 0.1)	1.0	>15*
				a	0.4 (0.3, 0.6)	1.0	>15*
y ~				Sugar intake ~			
(r_m^2 = 0.022, r_c^2 = 0.78)				(r_m^2 = 0.1, r_c^2 = 0.64)			
Partner diversity	-0.1 (-0.3, 0)	0.65	1.2	Partner diversity	0.03 (-0.04, 0.3)	0.29	-1.8
Interaction strength	0.01 (-0.1, 0.2)	0.26	-2.1	Interaction strength	-0.02 (-0.2, 0.06)	0.28	-1.9
Migratory distance	0.1 (0, 0.4)	0.61	0.91	Migratory distance	0.005 (-0.09, 0.1)	0.19	-2.9
Period	0.01 (-0.5, 0.6)	0.45	-0.43	Period	0.06 (-0.4, 0.6)	0.49	-0.084
Migratory dist. × period	0.01 (-0.05, 0.2)	0.10	-0.46	Migratory dist. × period	0.06 (-0.2, 0.5)	0.44	3.4*
				x	-0.3 (-0.7, 0)	0.84	3.4*
				y	-0.2 (-0.5, 0.003)	1.0	13*
				z	-0.3 (-0.6, 0.1)	1.0	>15*
				a	-0.4 (-0.6, -0.2)	1.0	12*
z ~				Protein intake ~			
(r_m^2 = 0.019, r_c^2 = 0.61)				(r_m^2 = 0.047, r_c^2 = 0.79)			
Partner diversity	0 (-0.09, 0.09)	0.18	-3.1	Partner diversity	-0.04 (-0.3, 0.04)	0.35	-1.3
Interaction strength	-0.01 (-0.2, 0.06)	0.22	-2.5	Interaction strength	0.003 (-0.1, 0.1)	0.19	-2.9
Migratory distance	-0.09 (-0.3, 0.03)	0.53	0.26	Migratory distance	-0.006 (-0.1, 0.07)	0.20	-2.8
Period	0.1 (-0.4, 0.7)	0.52	0.13	Period	-0.08 (-0.8, 0.4)	0.52	0.18
Migratory dist. × period	0.02 (-0.03, 0.3)	0.12	-0.12	Migratory dist. × period	-0.02 (-0.4, 0.3)	0.38	2.9*
				x	0.1 (0, 0.5)	0.51	0.11
				y	0.3 (0.1, 0.5)	0.99	8.8*
				z	0.04 (-0.1, 0.4)	0.31	-1.6
				a	0.007 (0, 0.1)	0.087	-4.7
a ~				Anthocyanin intake ~			
(r_m^2 = 0.18, r_c^2 = 0.66)				(r_m^2 = 0.5, r_c^2 = 0.86)			
Partner diversity	0.4 (0.2, 0.5)	1.0	>15*	Partner diversity	0.08 (0, 0.2)	0.71	1.8
Interaction strength	-0.01 (-0.2, 0.07)	0.22	-2.5	Interaction strength	0.01 (-0.01, 0.1)	0.23	-2.5
Migratory distance	-0.3 (-0.5, 0)	0.92	5.0*	Migratory distance	0.003 (-0.01, 0.07)	0.13	-3.8
Period	0.2 (-0.3, 0.9)	0.61	0.91	Period	-0.1 (-0.6, 0.3)	0.56	0.47
Migratory dist. × period	0.03 (-0.1, 0.4)	0.23	1.4	Migratory dist. × period	0 (-0.2, 0.2)	0.28	2.0*
				x	0.4 (0.1, 0.6)	0.98	8.2*
				y	0.3 (0.2, 0.5)	1.0	>15*
				z	0.6 (0.3, 0.8)	1.0	>15*
				a	-0.7 (-0.9, -0.6)	1.0	>15*

The structural equation model tested for direct and indirect effects of the partner diversity and interaction strength of frugivores in the networks, as well as their migratory distance, period (migration versus non-migration) and its interaction with migratory distance on the colour profile of consumed fruits (i.e., chromatic colour components (*x*, *y*, *z*) and the brightness (*a*) in avian colour space; see Methods) and on the mean intake of particular nutrients (i.e., lipid, sugar, protein, anthocyanin). The sample size was $n_{\text{obs}} = 165$ observations across $n_{\text{species}} = 43$ bird species, $n_{\text{site}} = 3$ study sites and $n_{\text{time}} = 4$ seasons. Animal phylogeny, species, site and season were included as random factors. Given are posterior means (with shrinkage), 95% credible intervals (CI), selection probabilities (P) and $2\log_e$(Bayes factor) (BF) as a measure of support for a given effect. Effects that were supported by the variable selection with BF > 2 are highlighted with an asterisk. The r^2 values depict the marginal (r_m^2) variance explained by fixed factors only as well as the conditional (r_c^2) variance explained by fixed and random factors combined[4]. Note that the results are consistent with the results based on the model in which the migratory distance outside the migration and pre-migration period was set to zero (see Table 2 in main text).

Supplementary Table 6. Summary of convergence statistics for Bayesian hierarchical model testing for relationships between fruit colour and the nutrient content of fruits.

Predictor	Response	PSRF	N_{eff}
x	lipid	1.005	2211
y	lipid	1.007	2186
z	lipid	1.005	1863
a	lipid	1.001	1852
x	sugar	1.006	2053
y	sugar	1.003	1949
z	sugar	1.002	2052
a	sugar	1.007	2129
x	protein	1.006	2225
y	protein	1.002	1810
z	protein	1.003	2071
a	protein	1.013	2071
x	anthocyanin	1.001	2053
y	anthocyanin	1.004	2102
z	anthocyanin	1.013	1840
a	anthocyanin	1.006	1950

The model tested the relationships between the chromatic colour components (*x*, *y*, *z*) and the brightness of fruits (*a*) in the avian colour space (see Methods for details) and the lipid, sugar, protein and anthocyanin concentrations in the fruit pulp (see Table 1 in main text). Plant phylogeny was included as a random factor. The sample size was $n_{species} = 44$ plant species. Given are pairs of predictor and response variables along with the potential scale reduction factor (PSRF) and effective sample size (N_{eff})[5]. Values of PSRF < 1.1 indicate that MCMC chains have converged on the same posterior distribution. N_{eff} indicates approximate sample size of posterior samples after accounting for temporal autocorrelation between posterior samples.

Supplementary Table 7. Summary of convergence statistics for Bayesian hierarchical structural equation models testing whether the fruit choice of frugivorous birds is mediated by fruit colour and whether the birds' mean intake of particular nutrients is related to their partner diversity, interaction strength and migratory behaviour.

		(**a**) Model in Table 2		(**b**) Model in Supplementary Table 3		(**c**) Model in Supplementary Table 4		(**d**) Model in Supplementary Table 5	
Predictor	Response	PSRF	N_{eff}	PSRF	N_{eff}	PSRF	N_{eff}	PSRF	N_{eff}
Partner diversity	*x*	1.007	2157	1.003	1897	1.003	2038	1.000	2033
Interaction strength	*x*	1.008	1965	1.000	2072	1.006	1961	1.004	1829
Migratory distance	*x*	1.006	1988	1.002	2064	1.009	2089	1.009	1917
Period	*x*	-	-	-	-	-	-	1.003	2000
Mig. distance × period	*x*	-	-	-	-	-	-	1.013	2197
Partner diversity	*y*	1.022	1906	1.004	2052	1.002	2371	1.002	1857
Interaction strength	*y*	1.011	2000	1.002	2189	1.005	1971	1.007	2224
Migratory distance	*y*	1.005	1940	1.006	1928	1.003	2033	1.000	1753
Period	*y*	-	-	-	-	-	-	1.012	2048
Mig. distance × period	*y*	-	-	-	-	-	-	1.021	1795
Partner diversity	*z*	1.008	2302	1.009	2110	1.007	2000	1.004	2379
Interaction strength	*z*	1.003	1802	1.003	2105	1.007	1956	1.008	1990
Migratory distance	*z*	0.999	2059	1.003	2308	1.006	1770	1.003	2000
Period	*z*	-	-	-	-	-	-	1.000	2000
Mig. distance × period	*z*	-	-	-	-	-	-	1.002	1933
Partner diversity	*a*	1.006	2160	1.003	1938	1.003	2000	1.026	2000
Interaction strength	*a*	1.012	1962	1.019	1740	1.003	2000	1.010	1934
Migratory distance	*a*	1.003	1947	1.013	1987	1.006	2161	1.025	2035
Period	*a*	-	-	-	-	-	-	1.002	2103
Mig. distance × period	*a*	-	-	-	-	-	-	1.001	1979
Partner diversity	lipid	1.003	1943	1.001	2091	1.000	1974	1.010	1981
Interaction strength	lipid	1.002	1811	1.002	1845	1.003	1820	1.013	2052
Migratory distance	lipid	1.005	2144	1.005	2170	1.013	2205	1.005	2092
Period	lipid	-	-	-	-	-	-	1.002	2073
Mig. distance × period	lipid	-	-	-	-	-	-	1.008	2065
x	lipid	1.004	2000	1.007	2469	1.003	2077	1.009	1881
y	lipid	1.003	1894	1.003	2141	1.005	2069	1.016	1954
z	lipid	1.003	1811	1.003	2000	1.000	2293	1.003	2116
a	lipid	1.001	1863	1.003	2077	1.002	2000	1.009	1891
Partner diversity	sugar	1.004	2000	1.004	2095	1.000	1923	1.012	1808
Interaction strength	sugar	1.006	1952	1.002	2028	1.002	2030	1.004	1911
Migratory distance	sugar	1.012	2000	1.015	2357	1.004	2069	1.003	1978
Period	sugar	-	-	-	-	-	-	1.005	1849
Mig. distance × period	sugar	-	-	-	-	-	-	1.000	2123
x	sugar	1.001	2023	1.006	1960	1.027	2098	1.010	1833
y	sugar	1.004	2009	1.009	1956	1.004	2224	1.009	2117
z	sugar	1.004	2119	1.009	2014	1.015	2056	1.008	2133
a	sugar	1.008	2143	1.008	2383	1.005	1996	1.008	1945
Partner diversity	protein	1.002	2314	1.006	1958	1.005	1841	1.004	1831
Interaction strength	protein	1.007	2546	1.009	2231	1.004	1940	1.004	2198
Migratory distance	protein	1.005	2043	1.006	1992	1.011	2085	1.004	1870
Period	protein	-	-	-	-	-	-	1.010	1957
Mig. distance × period	protein	-	-	-	-	-	-	1.002	2030
x	protein	1.001	2116	1.002	2055	1.009	2009	1.014	1932
y	protein	1.002	2312	1.013	2064	1.005	2139	1.004	2068
z	protein	1.003	1990	1.002	1908	1.013	2000	1.017	1935
a	protein	1.004	1924	1.012	1944	1.005	2175	1.004	2000
Partner diversity	anthocyanin	1.009	1892	1.001	1914	1.001	2320	1.002	2000
Interaction strength	anthocyanin	1.007	1852	1.003	1842	1.003	2048	1.012	2000
Migratory distance	anthocyanin	1.006	1947	1.001	2000	1.005	1992	1.005	1932
Period	anthocyanin	-	-	-	-	-	-	1.008	2165
Mig. distance × period	anthocyanin	-	-	-	-	-	-	1.009	1967
x	anthocyanin	1.003	2008	1.002	1890	1.001	2457	1.001	2345
y	anthocyanin	1.003	1966	1.003	2099	1.009	1944	1.002	2111
z	anthocyanin	1.004	2041	1.003	1906	1.000	2203	1.003	2219
a	anthocyanin	1.005	1933	1.001	1922	1.004	1761	1.001	1992

The models in (**a-d**) tested for direct and indirect effects of the partner diversity and interaction strength of frugivores in the networks and their migratory distance on the colour profile of consumed fruits (i.e., chromatic colour components (*x*, *y*, *z*) and the brightness (*a*) in avian colour space; see Methods) and on the mean intake of particular nutrients (i.e., lipid, sugar, protein, anthocyanin). Each model was based on different assumptions (see the summary table of each model for details). The sample size was $n_{obs} = 165$ observations across $n_{species} = 43$ bird species, $n_{site} = 3$ study sites and $n_{time} = 4$ seasons. Animal phylogeny, species, site and season were included as random factors. Given are pairs of predictor and response variables along with the potential scale reduction factor (PSRF) and effective sample size (N_{eff})[5]. Values of PSRF < 1.1 indicate that MCMC chains have converged on the same posterior distribution. N_{eff} indicates approximate sample size of posterior samples after accounting for temporal autocorrelation between posterior samples.

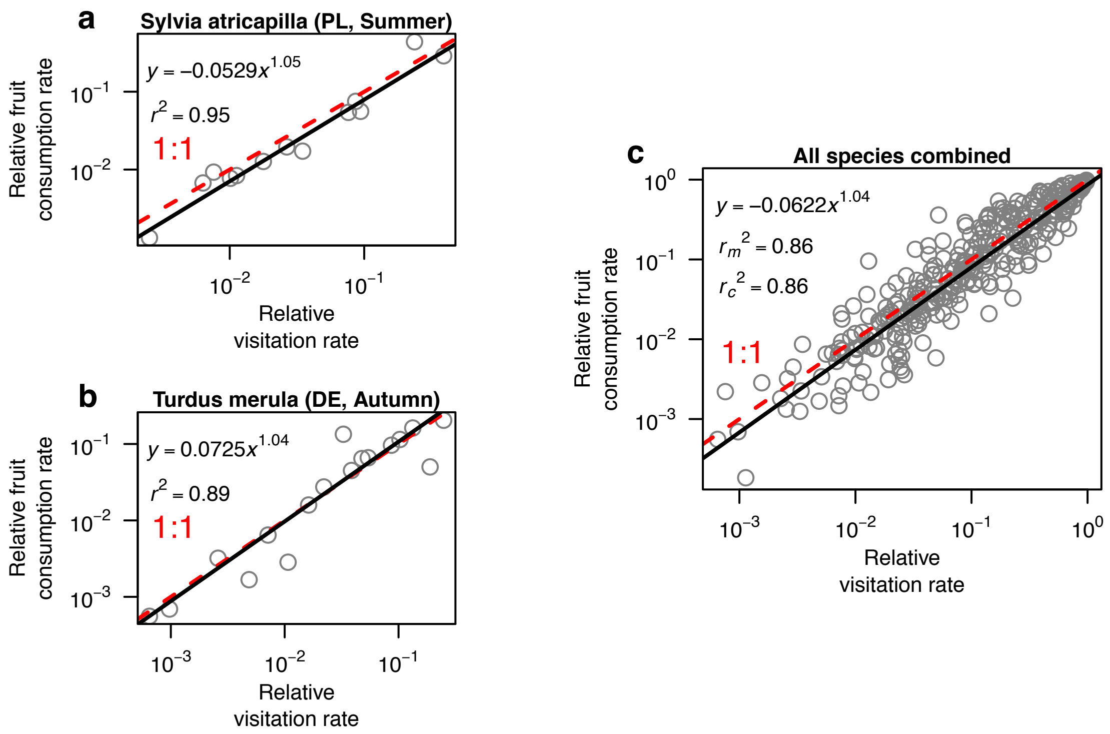

Supplementary Figure 1. Relationship between relative visitation and relative fruit consumption rate of frugivores on different plant species per hour based on data from Albrecht *et al.*[1] and Stiebel & Bairlein[2] for which fruit consumption rates per visit were available. (**a**,**b**) Example relationships between the relative visitation and relative fruit consumption rate shown for (**a**) *Sylvia atricapilla* (blackcap) in a summer network from Poland (PL) and (**b**) *Turdus merula* (blackbird) in an autumn network from Germany (DE). Sample sizes in (**a**,**b**) are $n_{\text{species}} = 13$ and $n_{\text{species}} = 17$ plant species on which the two bird species were recorded during foraging, respectively. The black lines in (**a**,**b**) are the estimated relationships from simple linear regressions of the form $\log(y) \sim a + b \times \log(x)$, where x is the relative visitation rate of the frugivore species on the plant species in a given network, y is the relative fruit consumption rate on these plant species, a is the intercept and b is the slope. (**c**) Relationship between the relative visitation and relative fruit consumption rate based on all frugivore species. The black line in (**c**) is the estimated relationship from a linear mixed effects model of the form: $\log(y) \sim a + b \times \log(x)$ + (1|frugivore species) + (1|network), where x, y, a and b are defined as above and frugivore species and network id are random factors. The sample size in (**c**) was $n_{\text{obs}} = 313$ observations across $n_{\text{species}} = 28$ frugivore species and $n_{\text{network}} = 6$ networks. Only frugivore species that were recorded on at least two plant species in a given network were included in the analysis. The r^2 values in (**c**) depict the marginal (r_m^2) variance explained by fixed factors only as well as the conditional (r_c^2) variance explained by fixed and random factors combined[4]. Note that the estimated relationship between relative visitation and fruit consumption rate per hour based on all frugivore species in (**c**) is statistically indistinguishable from a 1:1 relationship (i.e., the intercept does not differ from zero: $a = -0.062$, $z = -0.77$, $n = 313$, $P = 0.44$; and the slope does not differ from one: $b = 1.04$, $z = 1.49$, $n = 313$, $P = 0.14$). This indicates that the relative contribution of a plant species to the diet of a frugivore species is the same regardless of whether fruit consumption or visitation rates per hour are used to estimate interaction frequency. This is due to the fact that the comparatively large variation in the visitation rates of a frugivore species across different plant species overrides the comparatively small variation in the fruit consumption rate per visit of that frugivore species on each of the plant species[6]. The red dashed line in (**a**,**b**,**c**) represents the 1:1 line.

Supplementary References

1. Albrecht, J. *et al.* Variation in neighbourhood context shapes frugivore-mediated facilitation and competition among co-dispersed plant species. *Journal of Ecology* **103,** 526–536 (2015).
2. Stiebel, H. & Bairlein, F. Frugivorie mitteleuropäischer Vögel I: Nahrung und Nahrungserwerb. *Vogelwarte* **46,** 1–23 (2008).
3. Snow, B. K. & Snow, D. *Birds and berries: a study of an ecological interaction*. (T & A D Poyser, 1988).
4. Nakagawa, S. & Schielzeth, H. A general and simple method for obtaining R^2 from generalized linear mixed-effects models. *Methods in Ecology and Evolution* **4,** 133–142 (2013).
5. Plummer, M., Best, N., Cowles, K. & Vines, K. CODA: convergence diagnosis and output analysis for MCMC. *R News* **6,** 7–11 (2006).
6. Vázquez, D. P., Morris, W. F. & Jordano, P. Interaction frequency as a surrogate for the total effect of animal mutualists on plants. *Ecology Letters* **8,** 1088–1094 (2005).
