# Supplementary material for: Reward regulation in plant–frugivore networks requires only weak cues
Source: Nat Commun. 2018 Nov 16;9:4838. doi: 10.1038/s41467-018-07362-z (PMC6240120; doi:10.1038/s41467-018-07362-z)
Supplement: Supplementary file 3 — Description of Additional Supplementary Files [file 41467_2018_7362_MOESM3_ESM.pdf]

Description of Additional Supplementary Files

File Name: Supplementary Dataset 1
Description: JAGS model code. Monitored variables were the intercepts (alpha1, alpha2, alpha3); the standardized effect sizes of the explanatory variables (beta1, beta2, beta3); indicator variables denoting whether an explanatory variable has been selected or not (gamma1, gamma2, gamma3); and marginal and conditional variance explained by the models (rSq1, rSq2, rSq3). # precede comments.
